# Adapting wellbeing research tools for Aboriginal and Torres Strait Islander people with chronic kidney disease

**DOI:** 10.1186/s12882-020-01776-y

**Published:** 2020-04-15

**Authors:** Tricia Nagel, Michelle Sweet, Kylie M. Dingwall, Stefanie Puszka, Jaquelyne T. Hughes, David J. Kavanagh, Alan Cass, Kirsten Howard, Sandawana W. Majoni

**Affiliations:** 1 Menzies School of Health Research, Charles Darwin University, PO Box 41096, Casuarina, NT 0811 Australia; 2 Division of Medicine, Royal Darwin Hospital, Darwin, NT 0811 Australia; 3 Centre for Children’s Health Research, Institute of Health & Biomedical Innovation and School of Psychology & Counselling, Faculty of Health, Queensland University of Technology (QUT), Brisbane, QLD 4101 Australia; 4 Sydney School of Public Health, Faculty of Medicine and Health, University of Sydney, Sydney, NSW 2006 Australia; 5 Top End Renal Services, Royal Darwin Hospital, Northern Territory Department of Health, Darwin, NT 0810 Australia; 6 Northern Territory Medical Program, Flinders University, Darwin, NT 0815 Australia

**Keywords:** Indigenous wellbeing, E-mental health, Chronic kidney disease

## Abstract

**Background:**

Chronic kidney disease is an increasingly common health problem for Aboriginal and Torres Strait Islander people. It is associated with multiple concurrent psychosocial stressors frequently resulting in negative impacts on emotional and social wellbeing. There is need for well-designed intervention studies to provide evidence of effective treatment for comorbid depression or other mental illness in this setting. Attention to early phase piloting and development work is recommended when testing complex interventions. This paper documents feasibility testing and adaptation of an existing culturally responsive brief wellbeing intervention, the Stay Strong App, and three commonly used wellbeing outcome measures, in preparation for a clinical trial testing effectiveness of the intervention.

**Methods:**

The Stay Strong App, which has not been used in the setting of Chronic Kidney Disease before, is reviewed and adapted for people with comorbid wellbeing concerns through expert consensus between research team and an Expert Panel. The outcome measures (Kessler 10, Patient Health Questionnaire 9, and EuroQoL) are valid, reliable, and commonly used tools to assess various aspects of wellbeing, which have also not been used in this context before. Feasibility and acceptability are examined and developed through 3 stages: Pilot testing in a purposive sample of five haemodialysis patients and carers; translation of outcome measures through collaboration between the Aboriginal Interpreter Service, Aboriginal and Torres Strait Islander research officers and the research team; and conversion of translated outcome measures to electronic format.

**Results:**

Research team and expert panel consensus led to adaptation of the Stay Strong App for renal patients through selective revision of words and images. Pilot testing identified challenges in delivery of the wellbeing measures leading to word changes and additional prompts, integration of audio translations in 11 local Indigenous languages within an interactive Outcome Measures App, and related research protocol changes.

**Conclusion:**

Modelling the complex intervention prior to full-scale testing provided important information about the design of both the outcome measures and the intervention. These changes are likely to better support success in conduct of the clinical trial and future implementation of the intervention in clinical settings.

## Background

The health and welfare of Aboriginal and Torres Strait Islander Australians is improving in many areas, including life expectancy, educational attainment and child mortality [1]. Furthermore, a significantly higher proportion of Aboriginal and Torres Strait Islander people aged 15 and over reported good health in 2012–13 (37%) than in 2001 (33%). However, chronic kidney disease (CKD) is a serious and increasingly common health problem for Aboriginal and Torres Strait Islander people, especially those who live in remote communities. The most recent National Biomedical Risk Factor Survey (2012–13), estimated that while 10% of Australians have biomedical signs of CKD, a higher proportion (23%) of Aboriginal and Torres Strait Islander adults were estimated to have CKD, with a heavier burden of 39% of adults affected in remote areas [2]. CKD is categorised into five stages according to kidney function and evidence of kidney damage. End-stage renal disease (ESRD) or CKD-5 is the most severe form of CKD, where renal dialysis or kidney transplant is required for survival [3]. In 2012–13 the incidence rate of CKD-5 among Aboriginal and Torres Strait Islander Australians was 6.6 times that for non-Aboriginal and Torres Strait Islander Australians, and Aboriginal and Torres Strait Islander Australians were 10 times as likely as non-Aboriginal and Torres Strait Islander Australians to be hospitalised for this disease [4].

People with CKD sustain many losses - physical functions, cognitive abilities, and role in the family and workplace [5], and depression is common in those undergoing dialysis (25% when assessed by clinical interview, 40% when assessed by self-report measures) [6]. Depressive symptoms increase the risk of poor outcomes in people with ESRD on dialysis [7]. A Central Australian qualitative study describes the intense loneliness and social isolation of haemodialysis treatment as a prominent factor in missed treatment attendance [8, 9]. Most Aboriginal and Torres Strait Islander people with ESRD on dialysis in the Northern Territory of Australia have to relocate several hundred kilometres from their remote and very remote communities to access centrally based assisted-haemodialysis which is required thrice weekly [10]. This results in dislocation and social isolation, and the possibility that personal circumstances deteriorate further with time and disease progression [11].

Recent policy changes have enabled funding for renal nurses and Aboriginal Health Practitioners working in remote dialysis services [12]. Despite this investment, most renal patients from remote communities will continue to be treated in urban centres in the foreseeable future. The psychosocial implications of ESRD, compounded by the separation of patients from their communities and families, requires attention and targeted interventions. Evidence of effective treatment for depression or other mental illness in Aboriginal and Torres Strait Islander people (with or without co-occurring disorders) however, is difficult to find. Despite recognition that psychosocial factors are associated with morbidity and mortality in many chronic conditions, including CKD, well-designed intervention studies are lacking [5, 7]. One relevant intervention for consideration in this setting is the AIMhi Stay Strong App.

The Stay Strong App is the electronic version of one of the only formally evaluated, culturally responsive, mental health interventions for Aboriginal and Torres Strait Islander people. It was developed through the Northern Territory (NT) Aboriginal and Islander Mental Health Initiative (AIMhi). This brief intervention was designed in collaboration with local Aboriginal mental health workers (AMHW) through exploration of local Aboriginal and Torres Strait Islander perspectives of mental health [13, 14]. It combines elements of problem solving therapy and motivational interviewing, to create a ‘low-intensity’ treatment utilising a holistic, strengths-based approach with pictorial tools. The paper-based intervention was translated into tablet application format in 2013 with subsequent evaluation providing evidence of feasibility and acceptability [15].

Testing of a wellbeing intervention also requires the use of appropriate and psychometrically sound measures. Kessler Distress Scale (K^− 10^), Patient Health Questionnaire (PHQ-9) and EuroQoL (EQ-5D) are valid, reliable, and commonly used tools. However, these tools have not as yet been used to measure outcomes for Aboriginal and Torres Strait Islanders with CKD. K-10 is a measure of psychological distress with strong links between high scores and anxiety and depression. It is one of the routine outcome measures used by Australian public mental health services and has been used in full and abbreviated forms in state and nation-wide Aboriginal and Torres Strait Islander health surveys. It is chosen as the primary outcome measure in the absence of a robust and culturally responsive ‘wellbeing’ measure. PHQ-9 assesses severity of depression and has shown diagnostic, criterion and construct validity [16]. It has been tested with Aboriginal and Torres Strait Islander groups and adapted to include plain English response categories [17, 18] with additional specific adaptation for the Central Australian context [19]. Although there is overlap between the PHQ 9 and the Kessler 10, they are also complementary with the broader measurement of ‘emotional distress’ through use of the Kessler 10 complemented by the greater specificity of the PHQ 9 in measuring ‘depression’. The EQ-5D is a widely utilised multi-attribute utility instrument used for estimating utility weights for calculation of QALYs [20]. Although important work has been undertaken in Central Australia and nationally to adapt the English version of the PHQ-9 into Aboriginal English, none of the above outcome measures have been translated to Aboriginal and Torres Strait Islander languages [19, 21]. This is particularly relevant to research in the NT setting where most Aboriginal and Torres Strait Islander people speak English as a second or third language [22, 23].

In recognition that there are specific difficulties in defining, developing, documenting, and reproducing complex interventions that are subject to more variation than a drug, the Medical Research Council, developed a Framework for the Development and Evaluation of RCTs for Complex Interventions to Improve Health [24]. The framework recognises the specific difficulties in testing complex interventions, which may involve numerous interacting components within experimental and control interventions, and challenging protocol requirements for those delivering the intervention. In particular, the updated guidelines recommend greater attention to early phase piloting and development work, and recognition that complex interventions may work best if they are tailored to local contexts [25]. They emphasise that qualitative testing through focus groups, preliminary surveys, or case studies can help to define relevant components. This present study describes use of multimethod design to pilot and adapt outcome measures and the Stay Strong App prior to commencement of a clinical trial testing effectiveness. Such development work can also assist in rendering the tools appropriate, respectful, and relevant to the study population [26].

## Methods

### Study design and ethical approval

A multi-method approach was used for adaptation of the Stay Strong App intervention to the local context, and for pilot testing and further development of the outcome measures. Ethical approval for the research program was granted by relevant ethics committees (ref HREC 12–1881 and CAHREC 12–100,) including an Aboriginal sub-committee.

### Research team and expert panel

The 8-member research team comprised five non-Indigenous members with expertise in mental health and kidney health research in Aboriginal and Torres Strait Islander settings, a Torres Strait Islander renal physician and research fellow, and two Aboriginal and Torres Strait Islander research officers, one of whom spoke five Central Australian Aboriginal languages. A 9-member expert panel was established, consisting of two renal physicians, a renal dietician, four renal health nurses, one of whom is also Chief Executive Officer of Purple House **(**an Indigenous-owned and operated dialysis service based in Alice Springs), a cultural consultant and Aboriginal Elder from Central Australia, and a renal transplant recipient. The expert panel assisted the research team in adaptation of the Stay Strong App for renal patients. The research team also worked in collaboration with the Northern Territory Government Aboriginal Interpreter Service (AIS), which has offices in Darwin and Alice Springs and employs approximately 30 interpreters. The service provides interpreting and translation for the major languages of the Northern Territory and employs a further 400 casual interpreters covering nearly 100 languages and dialects.

### Adaptation of the stay strong app

The Stay Strong App has five sections: review of family, strengths, worries, and tips for wellbeing prior to setting life style goals for change. The expert panel reviewed the app and proposed changes. For example, given the specific dietary needs of renal patients some of the dietary suggestions within the app required adaptation. The recommended changes were then presented to the research team (Table 1). Further consultation within the team, the panel and with Aboriginal research team members led to revision of wording and images until consensus was reached.

**Table 1:** Stay strong app changes for aboriginal and Torres strait islander people with chronic kidney disease

Original wording or image	Revised wording or image
**Strengths**	
Good tucker	Healthy food
Spirituality	Strong spirit
Think positive	Think happy
Exercise	Change image representing football to one which shows activities around the house or going for a walk.
Music or Dance	Change icon to man and woman with painted bodies
Missing culture and country	Change icon to man and woman with painted bodies and picture of landscape
Having goals	Music and Dance
**Worries**	
Unhealthy lifestyle	Combine icons to show both unhealthy food and inactivity
Physical Illness	Sickness / Being sick
Anxious and Sadness	Worried or sad
**Stay strong tips**	
Eat Fruit and Vegie	Understand what healthy diet works for you - talk to dietitian
Drink Water	
Make new friends and do new things	Use time wisely
Additional prompt	Attend appointments / clinics / dialysis
Additional prompt	Talk to your care team

### Pilot testing of outcome measures

The chosen outcome measures (Kessler 10, Patient Health Questionnaire 9, and EuroQoL) were examined and adapted through 3 stages: pilot testing of feasibility and acceptability in a purposive sample of five haemodialysis patients and carers; translation of outcome measures through collaboration between the Aboriginal Interpreter Service, Aboriginal and Torres Strait Islander research officers and the research team into 11 Aboriginal languages (Warlpiri, Arrernte, Luritja, Pitjantjatjara, Alayawara, Tiwi, Kriol, Yolngu Matha, Ngangikurranggurr, Murrinh Patha, Anindiliyakwa), and conversion of paper-based outcome measures to electronic format.

### Participants and setting

The Western Desert Nganampa Walytja Palyantjaku Tjutaku (WDNWPT) Aboriginal Corporation runs Purple House, which has its headquarters in Alice Springs and provides dialysis and support to Aboriginal and Torres Strait Islander people with ESRD. Pilot testing of the paper-based version of outcome measures along with the Stay Strong App was carried out in a purposive sample of haemodialysis patients and carers, who were opportunistically recruited while attending Purple House on the morning that pilot testing commenced. Criteria for inclusion were: identification as Aboriginal and Torres Strait Islander, ability and willingness to participate, self-assessed facility with English language (no funding was allocated for interpreters within the pilot testing), age of 18 years or more, provision of oral informed consent, and ineligibility for the later clinical trial. Those who were eligible for the pilot testing but not the later trial included haemodialysis patients usually living in remote communities who were visiting Alice Springs short term, as well as carers of haemodialysis patients.

### Data collection

Verbal informed consent was obtained by the local (non-Indigenous) research officer, using a pictorial, plain English flip chart and a plain English information sheet developed in collaboration with Aboriginal research officers that explained the objectives of the project and the confidential handling of their data. Ethics approval was obtained to gain verbal rather than written consent considering the expected rates of written English literacy in this population. The pilot testing process was divided into three parts: 1) completion of the three paper-based outcome measures 2) completion of the Stay Strong App intervention; 3) completion of a semi-structured interview exploring ease of use, appropriateness and relevance of each tool. Responses during the first two parts of the pilot testing process were entered on the paper-based version of the outcome measures and into the Stay Strong App. The semi-structured interview responses were audio-recorded, and participant comments, suggestions, questions and non-verbal responses were noted, and later summarized and grouped into categories and thematically analysed. Participants were asked about each questionnaire: “How was that one? Anything you didn’t like about that one? Any questions you didn’t like or were hard to answer/not relevant to you?”. Participants were then asked about the Stay Strong App: “How did you feel when going through the app? What did you like about it? What didn’t you like about it? Is there anything that we could change to improve the Stay Strong App for people on dialysis?”

## Results

### Stage 1: Pilot testing of outcome measures

Three haemodialysis patients and two carers (three females, two males aged between 51 and 60 years) who spoke English as a second or third language participated in the pilot testing process through two individual and one group interviews (with three participants).

The outcome measures were generally well received, but several changes were recommended. The 10-item K-10 measure offers response categories of five frequency levels related to the past 2 weeks while the nine-item PHQ-9 uses four frequency levels and is scored with reference to the last 2 weeks. The transition between the 4 week and 2-week time frames in the different scales was not easily followed by the participants and required further explanation. Two K-10 questions (relating to the experience of ‘*hopelessness*’ and ‘*worthlessness*’) elicited a lack of verbal response. The researcher intentionally paused to provide time for participants to reflect on the questions and avoided rushing the response or interpreting silence as lack of understanding. Often the silence represented contemplation from which an answer later emerged. This approach was valuable but contributed to the length of the process. Where problems with understanding were encountered, alternative wording was discussed. After alternative words in English were presented, the group of three discussed the concepts in a common Aboriginal language (Pitjantjatjara) for several minutes before reaching consensus about alternatives. Alternatives proposed to the term ‘hopeless’ included ‘*without hope*’ ‘*not feeling good, ‘waking up like there is no hope’, and ‘what’s the point of getting up*’. An alternative to the term ‘worthless’ was ‘*no one want to know you’*.

One PHQ-9 question (‘*Have you been talking slowly or moving around really slow*?’) required further explanation. In addition, the transition from five frequency response options in the K-10 to four in the PHQ-9 led participants to request the missing category (‘*some of the time’*) as a useful available option. The EQ-5D was the easiest questionnaire to administer and was understood with ease in part because of its immediate time frame (today) but possibly also related to user-friendly and holistic attributes gained through its extensive development process within the multi lingual and multidisciplinary EuroQol Group [27]. There was nevertheless some difficulty in distinction between the ‘*slight*’ ‘*moderate*’ and ‘*severe*’ response categories for some items with participants struggling to identify the difference between the three options.

Participants responded positively to the Stay Strong App intervention, for example: ‘*ewa* (yes) *other people would like it’*; ‘*when they see it (the people who keep me strong) on the app they’ll start talking*’; ‘*I think it’s really good’*. No specific changes to the app were recommended. Feedback about the process of completing outcome measures also included positive comments: ‘*good to answer’*; ‘*made me feel better’*; ‘*I really like that one, was good to talk about that’*. There were also indications that the process was somewhat arduous with comments such as: ‘*a lot to answer’*; ‘*too long’*; ‘*feel too tired’* with related body language noted during the session (standing up, walking away, or answering the phone). One participant suggested dividing the process into two separate sessions ‘*Maybe next time catch up again’*.

One other comment suggested the questions ‘*need more explaining … it’s different English and Aboriginal and Torres Strait Islander languages … the words around feelings*’. While another said, ‘*That was a lot to answer in one go … (but) good to ask how I feel … let it out … made me feel better’.* Distractions within the environment (noise, people and activity) also appeared to contribute to difficulty in attending throughout the assessments and intervention process. The researcher observed that fatigue or boredom appeared to relate to two issues: the length of the assessment process (influenced by the above-mentioned challenges of language and distractions within the environment), and repetition within the outcome measures. The repetition within the outcome measures occurs because there is considerable overlap between the PHQ-9 depression scale and the Kessler 10 scale. For example, both explore symptoms of depression using similar wording.

### Stage 2. Outcome measures forward translation

The research team undertook a four-step process of forward translation of the outcome measures. The *first step* involved determination of the 11 most widely spoken Aboriginal languages (including Kriol) in each of the two regions in which the research was undertaken through consultation between research team, and the Aboriginal Interpreter Service and service providers.

In a *second step*, a plain English version of each of the tools with some terms commonly used by Aboriginal people in the NT was developed by the Aboriginal and Torres Strait Islander research officers for review by the research team. The *third step* involved evaluation of the plain English versions by the research team reconciling the different perspectives with the emphasis on conceptual rather than linguistic equivalence (whilst recognising that exact equivalence in this context is not possible). This step relied especially upon the bilingual members of the team seeking to identify and resolve the inadequate expressions of the translation. Discrepancies between team member views as well as the local interpretation of the different terms were addressed, and the final drafts were forwarded to AIS for further input and translation (*the fourth step*).

As a *fifth step* the research team had planned to undertake back translation to English by an independent translator whose mother tongue is English, to ensure that the questions maintained face validity and original meaning. This was not possible due to time and resource constraints as the AIS translations were often delayed for several months due to the difficulty of sourcing local language speakers who are trained interpreters.

### Stage 3: Paper to electronic outcome assessments with guiding protocols

The pilot testing of the hard copy outcome measures identified three key areas of concern which were not fully addressed through language translation: difficulty explaining the time frames, difficulty understanding the terms ‘*worthless*’ and ‘*hopeless*’, and fatigue and boredom related to the assessment process and repetition within the outcome measures. Consultation within the research team with reference to the Aboriginal and Torres Strait Islander researchers led to changes to the process of administration of the outcome measures and to their presentation. Additional prompts for the time frames were developed to accompany the questions, which anchored the time frames in events with similar time patterns (e.g. the fortnightly schedule of wages and government payments and benefits) and the team confirmed that interpreters would be used to assist with these explanations. An additional prompt for ‘*worthless*’ was included: ‘*no one want to know you’*. Although prompts were added to protocols, only one word within the Kessler 10 and the PHQ-9 scales was ultimately changed. The term ‘*hopeless*’ was altered to feeling ‘*without hope*’. The associated explanatory prompts for this term were: ‘*not feeling good, waking up like there is no hope, what’s the point of getting up*’. In addition, the team confirmed that finding mutually agreed quiet places for the assessment, allowing participants time to generate their responses, allowing interviews to be broken up into consent, assessment and treatment sessions if required, and ensuring the presence of interpreters and Aboriginal and Torres Strait Islander research officers throughout would improve communication. In a final stage of outcome measure adaptation, the team collaborated with our App developer to develop a user-friendly electronic version of each tool with images and visual cues. AIS identified trained interpreters who were native speakers to record audio versions in Aboriginal and Torres Strait Islander languages. These were then forwarded to our App developer allowing both audio and written translation of the outcome measures with accompanying visual images for use via an electronic (i.e. tablet application) format.

### Finalised tools

The revised Stay Strong App has incorporated the recommended wording and image changes. The completed Outcome Measures App is in electronic tablet format. Each of the three outcome measures (K-10, PHQ-9 and EQ-5D) is supported by 11 language options with visual cues and optional audio files. Both tools were finalised in preparation for use as assessment and treatment tools within a clinical trial of effectiveness of the MCP intervention for chronic kidney disease patients.

## Discussion

By taking a phased approach to the development and evaluation of complex interventions through piloting and feasibility work, researchers can have greater confidence that the intervention can be delivered as intended both through the clinical trial and potential later implementation within routine care. This study found that miscommunication and fatigue were potential barriers to success. These challenges often arise when research protocols require multiple outcome measures to be completed in a structured and consistent manner. The difficulty is further complicated in the Northern Territory by the high numbers of Aboriginal language speakers, the multiple languages which are spoken by patients, the low number of service providers speaking Aboriginal languages, and the relatively few available interpreters. The study allowed potential challenges to be addressed through adaptation of tools and processes and confirmed the importance of working together with Aboriginal researchers, interpreters and community members to find solutions.

Time frames were one of the key concepts within the outcome measures that were not easily understood. This difference between Aboriginal and Torres Strait Islander and Western ways of measuring and anchoring time has been identified and described in detail in many other settings [28]*.* Difficulties with terms such as ‘hopelessness’ have also been encountered elsewhere. Brown et al. reported similar translational difficulties for bilingual experts who felt that ‘*the overarching equivalent for the term was the constellation of depressive feelings and therefore left hopelessness out of the PHQ-9 adaptation’* [19]. Key solutions proposed to deal with miscommunication and fatigue were embedded into the accompanying assessment protocols and included:

- Identifying mutually agreed quiet places for the assessment
- Allowing participants time to contemplate and generate their responses
- Allowing interviews to be divided into consent, assessment and treatment sessions if required
- Presence of interpreters and Aboriginal and Torres Strait Islander research officers wherever possible
- Identifying alternative prompting phrases to deal with items linked with difficulty in translation

The addition of the prompt of “*no one want to know you*” for the K10 “*worthless*” item may be considered to introduce a social aspect that is not explicitly in the original item. This clarification reflects an important aspect of many Indigenous cultures including Aboriginal and Torres Strait Islander people, where self-concepts are inextricably linked to family and community [29].

In recognition of the burden of symptoms accompanying end stage kidney disease, patient experience measures are under development internationally to inform patient care needs and clinical quality measures [30]. We have demonstrated that Western understanding of ESRD symptoms differ from Aboriginal peoples understanding of symptoms, but we have enabled a validation of Aboriginal-reported experience and symptom measures and scoring of measures in this study. This research is highly relevant to health services and patients, and will be transferable to quality audit and patient care for Aboriginal patients in this region [11].

Limitations of this study include the small sample recruited to this pilot testing, which limits confidence in concluding that the changes generalise to the broader population of NT Aboriginal and Torres Strait Islander people with ESRD on hemodialysis. In addition, the conduct of a group interview rather than individual interviews raises the possibility that members of the group influenced each other’s responses. On the other hand, the addition of the input of the expert panel, the Aboriginal Interpreter Service, the Aboriginal and Torres Strait Islander members of the research team, and the experience of the non-Indigenous members of the research team, provided additional perspectives to add confidence to the findings. The lack of back translation to English by an independent translator whose mother tongue is English, is another limitation of this study. This step will be important to undertake in the future when the research team is not facing time and resource constraints, to ensure that the questions maintained face validity and original meaning.

## Conclusion

Aboriginal and Torres Strait Islander people with Chronic Kidney Disease Stage 5 (CKD-5) face many wellbeing challenges. Their unique experiences require the development of targeted interventions supported by evidence of effectiveness obtained through robust research design. However, researchers must recognize that research practices and processes and related interventions are embedded in Western biomedical knowledge traditions; and may not translate into Aboriginal and Torres Strait Islander expectations of healthcare and research, ways of relating to people and broader ontologies of health and care. Key learnings from our process are that modelling the complex intervention prior to full scale testing with Aboriginal and Torres Strait Islander researchers, service providers and end users provided important information about the design of both the outcome measures and the intervention. The consequent changes are likely to better support success in conduct of the clinical trial and future implementation of the intervention in clinical settings. This study reports on the initial phase of preparation for a clinical trial seeking to find ethical, respectful and effective research strategies through translation and adaptation of the research tools and processes.

## Data Availability

The datasets generated and/or analysed during the current study are not publicly available due to participant confidentiality but may be available from the corresponding author on reasonable request.

## Abbreviations

AIMhi: Aboriginal and Islander Mental Health Initiative
AIS: Australian Interpreter Service
AMHW: Aboriginal mental health worker
CKD: Chronic kidney disease
CKD-5: Chronic Kidney Disease Stage 5
EQ5D: EuroQoL
ESRD: End Stage Renal Disease
K10: Kessler Psychological Distress Scale
NT: Northern Territory
PHQ-9: Patient Health Questionnaire
QoL: Quality of Life
QALY: Quality adjusted life years
RCT: Randomised controlled trial
WDNWPT: Western Desert Nganampa Walytja Palyantjaku Tjutaku Aboriginal Corporation
